# Revisiting Text-Based Person Retrieval: Mitigating Annotation-Induced Mismatches with Multimodal Large Language Models

**DOI:** 10.3390/s26051599

**Published:** 2026-03-04

**Authors:** Zihang Han, Chao Zhu, Mengyin Liu

**Affiliations:** School of Computer and Communication Engineering, University of Science and Technology Beijing, Beijing 100083, China; zihanghan@xs.ustb.edu.cn (Z.H.); mengyinliu@xs.ustb.edu.cn (M.L.)

**Keywords:** text-based person retrieval, annotation-induced mismatches, multimodal large language models

## Abstract

Text-based person retrieval (TBPR) aims to search for target person images from large-scale video clips or image databases based on textual descriptions. The quality of benchmarks is critical to accurately evaluating TBPR models for their ability in relation to cross-modal matching. However, we find that existing TBPR benchmarks have a common problem, which often leads to ambiguities where multiple images of persons with different identities have very similar or even identical textual descriptions. As a consequence, although TBPR models correctly retrieve the images corresponding to a given description, such matches may be erroneously evaluated as mismatches due to the above annotation problem. We argue that the main cause of this problem is that each person image is annotated individually without reference to other similar images, making it challenging to provide distinctive descriptions for each image. To address this problem, we propose an effective and efficient annotation refinement framework to improve the annotation quality of TBPR benchmarks and thereby mitigate annotation-induced mismatches. Firstly, sets of images prone to mismatches are automatically identified by TBPR models. Then, by leveraging multimodal large language models (MLLMs), multiple images are simultaneously processed and distinctive descriptions are generated for each image. Finally, the original descriptions are replaced to improve the annotation quality. Extensive experiments on three popular TBPR benchmarks (CUHK-PEDES, RSTPReid and ICFG-PEDES) validate the effectiveness of our proposed method for improving the quality of annotations, and demonstrate that the resulting more discriminative captions can truly benefit the mainstream TBPR models. The improved annotations of these benchmarks will be released publicly.

## 1. Introduction

With the rapid advancement of video surveillance and digital imaging technologies, text-based person retrieval (TBPR) has emerged as a significant research direction in the field of computer vision. This task aims to retrieve person images using textual descriptions (e.g., “a man in a red jacket carrying a black backpack” or “a woman wearing a white shirt and blue jeans”), with applications in areas such as video surveillance (e.g., quickly locating a suspect across city-wide camera networks) [1]. Unlike traditional image-based retrieval methods, text-based person retrieval requires models to identify the best-matching image for a given textual description from a large-scale image database. This necessitates the fine-grained cross-modal alignment between images and text [2], making the granularity of dataset annotations critically important.

In practical deployments, TBPR is tightly coupled with camera and vision sensors in large-scale surveillance and public security systems. The “person images” in TBPR benchmarks are in fact the outputs of networked camera sensors deployed in urban spaces, transportation hubs, campuses and commercial environments. These sensors continuously acquire visual data streams, which are then organized into large-scale, sensor-captured image or video databases. Text-based person retrieval builds a human–computer interaction layer on top of such sensor infrastructures, allowing operators to search for target individuals using natural language descriptions instead of exemplar images, and thus enabling more flexible and intuitive interaction with vision–sensor networks in time-critical scenarios such as suspect tracking, missing person search and post-event forensics. From this perspective, improving the reliability and discriminability of TBPR benchmarks directly benefits the design, evaluation and deployment of camera sensor-based intelligent surveillance systems.

Despite their widespread adoption, mainstream TBPR benchmarks such as CUHK-PEDES [1], RSTPReid [3] and ICFG-PEDES [4] are annotated at relatively coarse granularity, where each image is described in isolation and many identities share short, highly templated sentences. In practice, we observe groups of different identities (IDs) that even share exactly the same caption, implying that a single description can semantically match multiple persons and that correctly retrieved images may still be penalized as negatives during evaluation. This dataset-level ambiguity motivates us to revisit and refine the annotations of existing TBPR benchmarks.

**Figure 1:**
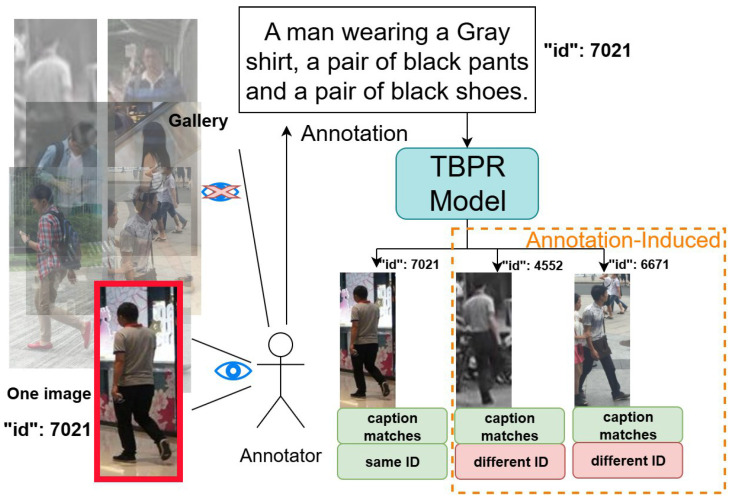
Illustration of annotation-induced mismatch in TBPR.

As illustrated in Figure 1, a key challenge arises when annotators, normally not comparing multiple similar images simultaneously, provide nearly identical descriptions to visually similar person with different IDs. During retrieval, although the model accurately matches all images that fit the description, those with different IDs are incorrectly treated as mismatches. This phenomenon, known as annotation-induced mismatch, reflects errors originating from the annotation process rather than model inference.

**Figure 2:**
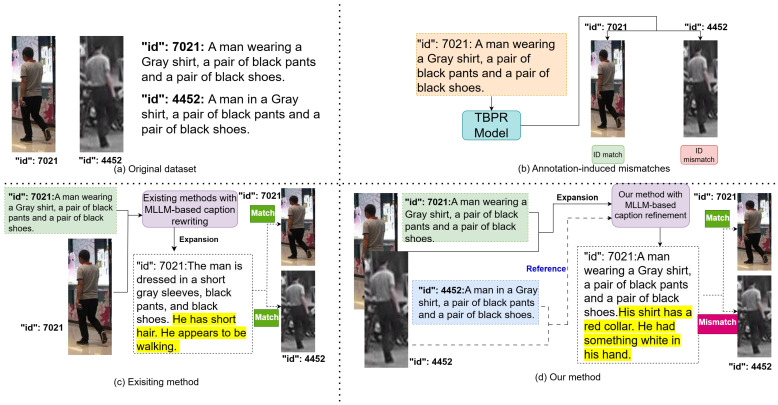
Comparison of our proposed method with the existing method for addressing annotation-induced mismatches.

Figure 2a shows two person images and their corresponding descriptions from the CUHK-PEDES benchmark [1]. Figure 2b illustrates an example of a mismatch: when the description corresponding to the left person image is input into the TBPR model, the model correctly retrieves two person images that match the description. However, since the right image has a different ID from the description, it is treated as a negative sample with respect to the description.

Existing works have attempted to enhance the granularity of dataset annotations [5,6] and have explored multimodal large language models (MLLMs) for data augmentation [7,8]. As illustrated in Figure 2c, these methods annotate individual images with more detailed and lengthy textual descriptions. However, these approaches do not allow the model to compare across images, meaning that even with more detailed captions, a single caption may still correspond to multiple images. We believe that the main cause of mismatches is that the annotators did not view multiple images simultaneously, making it challenging to provide distinctive descriptions for each image.

To address this issue, as shown in Figure 2d, we leverage MLLMs to compensate for the limitations of human annotators in observing multiple images at once. This approach reduces the likelihood that a single caption corresponds to multiple images with different IDs. Specifically, we propose an annotation refinement framework to identify image–text pairs in coarsely annotated datasets where the textual description corresponds to multiple images with different IDs.

We then incorporate MLLMs to enable the model to observe multiple images and their associated textual descriptions simultaneously. This allows for the generation of more detailed and discriminative captions. We propose a Separate Expansion Followed by Merging strategy to alleviate mismatches, where the texts in different sets of image–text pairs that could lead to mismatches are independently expanded, and then the expanded texts are merged into a modified dataset.

Finally, we validate our methods through a combination of model-agnostic and mainstream TBPR evaluations. On the modified portion of the CUHK-PEDES test set, we first adopt frozen vision–language models, ViT-bigG-14 (CLIP) and SigLIP-large, to perform zero-shot retrieval and compute discriminative capability metrics. After refinement, CLIP_R@K consistently improves, the MeanMargin between positive and hardest negative images becomes larger, and the proportions of ambiguous descriptions (Rate_margin <0 and Rate_margin <0.01) decrease, indicating that the refined captions are intrinsically more discriminative and better aligned with their corresponding images. In addition, to verify the generalizability and effectiveness of our annotation refinement strategy, we train and evaluate several representative TBPR models on the refined annotations on three popular TBPR benchmarks: CUHK-PEDES, RSTPReid [3] and ICFG-PEDES [4]. Across all three benchmarks, Rank@1 and mAP are generally improved, which can be viewed as supporting evidence that the retrieval performance of mainstream TBPR models also benefits from the refined annotations.

The main contributions of this paper are summarized as follows:

(1) **Annotation-induced mismatch in mainstream TBPR benchmarks.** We point out a common issue in mainstream TBPR datasets: a single textual description can correspond to multiple images with different IDs. A key reason is that annotators usually do not compare multiple similar images at the same time, which makes it difficult to provide distinctive descriptions for each image. This issue may introduce bias into evaluation and affect the objective assessment of model performance.
(2) **MLLM-based refinement by comparing multiple images simultaneously.** We address this issue by enabling the annotation process to consider multiple images simultaneously, rather than annotating each image independently. Specifically, we introduce MLLMs to automatically compare and analyze several visually similar images at once, and to generate more distinctive and discriminative textual descriptions for each image. Compared to purely manual annotation, this MLLM-based approach provides a more systematic and efficient way to reduce annotation-induced mismatches.
(3) **Discriminative capability evaluation with frozen vision–language models.** We quantitatively study the effect of the refined annotations through zero-shot evaluation with frozen ViT-bigG-14 (CLIP) and SigLIP-large on the modified portion of CUHK-PEDES. After refinement, CLIP_R@1 increases by 39.7% and 53.4%, respectively; the MeanMargin improves, and the proportions of ambiguous descriptions (Rate_margin <0 and Rate_margin <0.01) become lower. In line with these observations, four representative TBPR models evaluated on the refined test sets obtain higher Rank@1 on CUHK-PEDES, RSTPReid and ICFG-PEDES, which can be viewed as supporting evidence that the retrieval performance in standard TBPR settings also benefits.
(4) **Refined test sets released.** We refine the test sets of three popular benchmarks including CUHK-PEDES, RSTPReid and ICFG-PEDES, and release the improved annotations to facilitate further research in the field. The improved annotations of these benchmarks will be publicly available at https://github.com/hzh0720/Revisiting-Text-Based-Person-Retrieval, accessed on 2 March 2026.

## 2. Related Work

### 2.1. General Methods for Text-Based Person Retrieval

Text-based person retrieval (TBPR) typically learns a shared embedding space in which image and text features can be efficiently aligned for accurate retrieval. The task was formally introduced with the CUHK-PEDES benchmark, where a CNN-RNN pipeline (e.g., VGG for images and LSTM for text) was optimized by a matching loss to couple the two modalities [1]. Subsequent works upgraded both branches, adopting deeper vision backbones and stronger sentence encoders, and explored two-branch architectures that either learn a shared embedding or directly regress similarity [9]. On the text side, sentence-level transformers such as SBERT provided more discriminative and retrieval-friendly sentence embeddings [10].

Beyond backbone advances, global alignment objectives have been refined. Cross-Modal Projection Matching and its companion classification objective improve global image–text alignment by aligning projected distributions and strengthening class compactness [11]. Complementary to this, global semantic consistency has been coupled with attention-based local alignment to regularize cross-modal similarity learning [12].

To enhance discriminability, many methods introduce local feature branches for fine-grained alignment. Pose-guided multi-granularity attention selects body part regions and aligns them with phrases to capture structured correspondences [13]. Semantically self-aligned networks further extract part-aware features across modalities to reduce intra-class textual variance [4]. Surroundings–person separation explicitly isolates person information from background to suppress misaligned cues [3]. In parallel, implicit relation reasoning integrates visual cues into textual tokens and aligns distributions to strengthen both local and global matching [14]. More recently, Chen proposed Fine-grained Cross-modal Semantic Alignment (FCSA) [15], which strengthens fine-grained image–text matching by combining a Cross-Modal Reconstruction Strategy (CMRS) for bidirectional cross-modal reasoning with a Saliency-Guided Masking Mechanism (SGMM) that dynamically focuses on salient visual patches and critical text tokens, thereby improving robustness to subtle semantic variations in text-based person search.

Attention mechanisms are central to modeling local relations. Bi-attention designs combine intra-modal self-attention with inter-modal co-attention to capture holistic image–text correlations [16]. Graph-based formulations propagate complementary semantics among neighborhood samples to mitigate incompleteness and reduce modality gaps [17]. Adversarial objectives have also been employed to learn modality-invariant projections while preserving cross-modal structure [18].

Large-scale vision–language pre-training has reshaped TBPR. Contrastive Language–Image Pre-training (CLIP) supplies transferable encoders and a robust InfoNCE-style objective that generalizes well across a wide range of vision–language tasks [19]. Building on CLIP, recent studies establish strong TBPR baselines by re-examining data augmentation, loss design, and training practices [20].

Training objectives continue to evolve around hard negative discrimination and robustness. Margin-enforcing angular losses and pairwise weighting improve intra-class compactness and inter-class separability in the joint space [21]. RaSa introduces the relation-aware detection of strong versus weak positive pairs and the sensitivity-aware detection of word-level perturbations to improve robustness under noisy correspondences and augmentations [22]. Robust dual embedding strategies further address noisy correspondences by selecting clean pairs and relaxing triplet constraints to prevent collapse under misalignment [23]. Attribute-aided spaces provide additional semantic supervision to bridge modalities and enhance interpretability [24]. Meanwhile, unsupervised cross-modal hashing explores efficient large-scale retrieval via shared latent codes with graph-regularized factorization [25].

### 2.2. Enhancement of Textual Description

Obtaining large-scale, fine-grained textual descriptions remains a major bottleneck due to the cost of manual annotation. Early captioning pipelines leveraged detected attributes and template-based sentence generation to reduce labeling overhead, albeit with limited diversity and expressiveness [6]. New datasets with denser and longer descriptions, such as IIITD-20K, have been proposed to increase identity diversity and provide richer supervision for text-to-image ReID [26]. In a similar vein, UFineBench introduces ultra-fine-grained descriptions and an evaluation protocol that better reflects real-world granularity [5].

To scale annotation, multimodal large language models have been adopted to automatically generate captions. BLIP leverages bootstrapped captioning and filtering to create higher-quality supervision for retrieval and related tasks [27]. Instruction-tuned LVLMs such as LLaVA and Qwen-VL further enable general-purpose visual–language description and grounding with improved instruction following [28,29]. However, MLLM-generated captions can be stylistically homogeneous due to prompt or instruction bias. To address this, the HAM framework explicitly models human annotator styles by clustering real captions and prompting MLLMs to mimic diverse style prototypes, substantially improving description diversity and generalization [30].

Benchmarks and evaluation protocols are evolving with the data. Beyond CUHK-PEDES, datasets such as ICFG-PEDES and RSTPReid broaden domain diversity and supervision regimes for TBPR [3,4]. Standard metrics like Recall@K and mAP remain prevalent, while UFineBench further proposes mean Similarity Distribution (mSD) to sensitively assess fine-grained retrieval [5]. In parallel, robustness to noisy or weakly aligned text–image pairs has become increasingly important; recent methods explicitly detect clean pairs and relax hard negative constraints to maintain reliable training under imperfect supervision [23].

In summary, TBPR has evolved from basic global feature alignment to fine-grained, part-level, and robust matching frameworks, driving continuous progress in feature extraction, semantic alignment, and caption generation. However, despite efforts in previous works to improve annotation granularity by providing more detailed and lengthy textual descriptions, a single caption may still correspond to multiple similar images. We argue that the main cause of this problem is that it is hard for annotators to simultaneously compare multiple images, which makes it challenging to provide unique and discriminative descriptions for each image. In contrast, our refinement framework explicitly uses error groups and an MLLM to compare multiple similar images simultaneously and expand captions by emphasizing identity-specific visual differences, which directly mitigates annotation-induced mismatches that can bias benchmark evaluation.

## 3. Methodology

**Figure 3:**
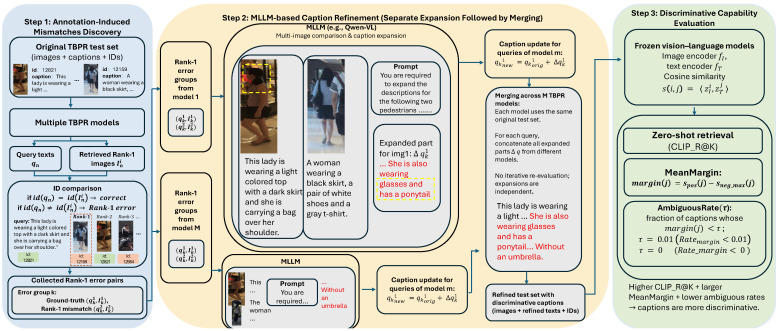
Overall workflow of the proposed annotation refinement framework. Step 1 discovers annotation-induced mismatches by collecting Rank-1 error pairs from multiple TBPR models on the original test set. Step 2 performs MLLM-based caption refinement with the Separate Expansion Followed by Merging strategy. Step 3 evaluates the discriminative capability of the refined captions using frozen vision–language models and representative TBPR models.

In this section, we introduce a three-step annotation refinement framework for text-based person retrieval (TBPR), as illustrated in Figure 3. Step 1 discovers annotation-induced mismatches by running several strong TBPR models on the original test set and collecting Rank-1 error pairs that reveal captions likely to describe multiple identities. Step 2 refines the captions of these error-prone image–text pairs with a multimodal large language model (MLLM) using a Separate Expansion Followed by Merging strategy so that each description becomes richer and more identity specific, and Step 3 evaluates the refined annotations with frozen vision–language models and mainstream TBPR models to verify that the new captions are more discriminative and support more reliable retrieval performance.

To make the workflow more concrete, Figure 3 also visualizes a typical example that runs through the three steps. In the CUHK-PEDES test set, a query caption “This lady is wearing a light-colored top with a dark skirt and she is carrying a bag over her shoulder” is associated with a particular image (the ground truth identity). When we apply one TBPR model, its Rank-1 retrieved image is another woman whose caption is “A woman wearing a black skirt, a pair of white shoes and a gray t-shirt.” Both pedestrians wear a dark skirt and a light-colored upper garment, so the short original caption can semantically describe both images, even though their IDs are different. In Step 1, this Rank-1 error is detected by the ID comparison and the two image–text pairs are grouped into an error group, where $\left(q_{k}^{1},I_{k}^{1}\right)$ denotes the ground truth image–text pair and $\left(q_{k}^{2},I_{k}^{2}\right)$ denotes the Rank-1 mismatched pair.

In Step 2, this error group is fed into the MLLM together with the discriminative prompt (e.g., “You are required to expand the descriptions for the following two pedestrians. Please pay attention to the differences between them and explicitly mention these differences in your expansions.”). By jointly inspecting the two similar images, the MLLM identifies details that distinguish the ground truth person from the confusing one. For instance, in the example shown in the center of Figure 3, the MLLM expands the ground truth caption with phrases such as “She is also wearing glasses and has a ponytail,” while another refinement (coming from a different TBPR model on the same query) adds that the woman is “walking without an umbrella.” After applying the proposed Separate Expansion Followed by Merging strategy, all these expanded segments are concatenated to the original caption, resulting in a refined description such as “This lady is wearing a light colored top with a dark skirt and she is carrying a bag over her shoulder. She is also wearing glasses and has a ponytail, and she is walking without an umbrella.” These additional, objective details increase the semantic separation between captions of different identities, reduce cross-identity overlap in the textual space, and ultimately make the refined dataset a more accurate and reliable benchmark for evaluating TBPR models. Because Step 1 relies on Rank-1 retrieval errors produced by existing TBPR models, one may worry about a circular dependency where model weaknesses determine which annotations are treated as “incorrect”. We clarify that in our framework a Rank-1 error is not regarded as ground truth evidence that a caption is semantically wrong; instead, it serves as a high-recall heuristic to surface candidate ambiguous cases where a short description can plausibly apply to multiple identities under the ID-based evaluation protocol (case (2) in Table 1). To reduce model-specific bias, we aggregate error groups from multiple heterogeneous TBPR models and avoid any iterative re-evaluation or re-training during candidate collection. The actual caption updates are generated by an external MLLM via direct cross-image comparison in Step 2, and the effectiveness is further verified by model-agnostic evaluations, including frozen vision–language diagnostics (Section 3.3) and human evaluation (Section 4.4).

### 3.1. Step 1: Annotation-Induced Mismatches Discovery

In TBPR tasks, textual descriptions may accurately correspond to images but are assigned different IDs. During model training or evaluation, even if the model correctly matches images to their descriptions, these pairs may be incorrectly classified as mismatches due to annotation errors in the dataset. This issue arises because, in the current annotation process, annotators typically observe and describe individual images without a holistic view of the entire image set. As a result, a caption created for one image may also apply to another image with a different ID.

**Figure 4:**
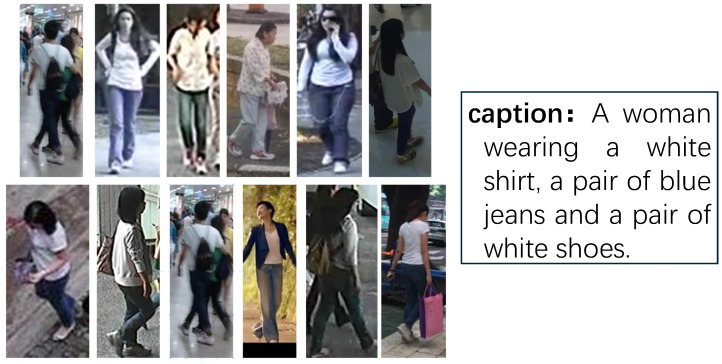
Visualization of an extreme case of coarse-grained annotation-induced mismatches. Multiple person images with different IDs share the same textual description, so a single caption can semantically match several images.

As shown in Figure 4, there exists an extreme case in the CUHK-PEDES dataset, where 12 person images with different IDs share the same caption. In reality, the caption “A woman wearing a white shirt, a pair of blue jeans, and a pair of white shoes” could accurately describe all 12 images. However, during testing, due to the different IDs of these images, only one image with the same ID will be considered a match.

In datasets related to the TBPR domain, each image is paired with one or more human-annotated descriptions. Our goal in this step is to locate those image–text pairs whose textual description can also accurately describe other person images with different IDs, so that they are prone to annotation-induced mismatches.

To formalize this, Table 1 enumerates all the possible scenarios between each textual description and image in the dataset. When a description accurately reflects the person depicted in the image, the image–text pair is considered semantically matched; otherwise, it is considered semantically mismatched. During evaluation, if the textual description and the image share the same ID, they are treated as a match; otherwise, they are treated as a mismatch. Within this classification framework, case (1) represents semantic matching that is also judged as a match during evaluation, which is correct. Case (2) corresponds to image–text pairs that are semantically matched but, due to different IDs, are judged as mismatched during evaluation—this is an inaccurate scenario and is exactly the type of potential annotation-induced mismatch we aim to address. Case (3) refers to semantically mismatched pairs that are incorrectly judged as a match due to sharing the same ID; such cases are expected to be rare and mainly caused by annotation errors. Case (4) represents semantic mismatches that are also judged as mismatches during evaluation, which is correct.

**Figure 5:**
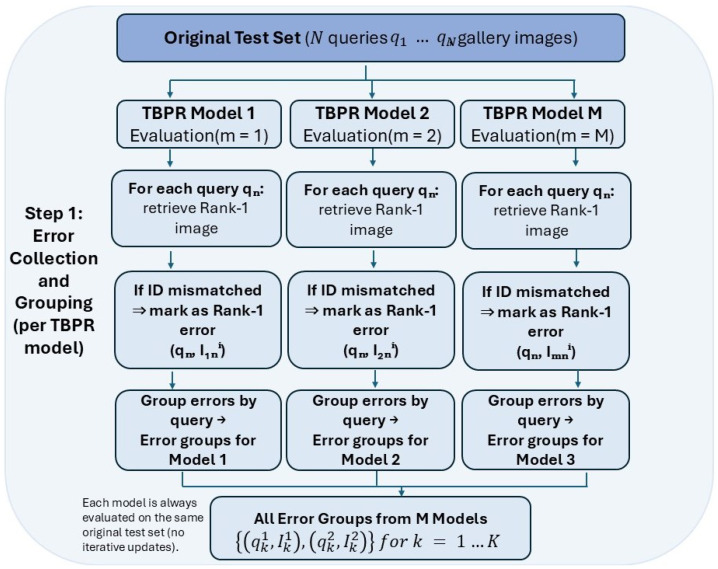
Step 1 of the Separate Expansion Followed by Merging strategy: error collection and grouping for each TBPR model. For every model, we evaluate it on the original test set once, mark Rank-1 mismatches by comparing person IDs, and then group all Rank-1 error pairs by query text. This yields per-model error groups that approximate annotation-induced mismatches.

However, it is impractical to manually screen all instances of case (2). Therefore, as illustrated in Figure 5, we adopt a more efficient procedure to automatically collect image–text pairs that are most likely to fall into case (2). Figure 5 visualizes Step 1 of our Separate Expansion Followed by Merging strategy, namely error collection and grouping per TBPR model.

Starting from the original test set at the top of Figure 5, we assume there are *N* query texts $\left\{q_{1},q_{2},\ldots ,q_{N}\right\}$ and a gallery of person images. We also consider *M* state-of-the-art TBPR models, $\left\{{\mathrm{TBPR}\_\mathrm{model}}_{1},{\mathrm{TBPR}\_\mathrm{model}}_{2},\ldots ,{\mathrm{TBPR}\_\mathrm{model}}_{M}\right\}$. Each model is evaluated independently on the same original test set (no iterative updates are performed at this stage, as emphasized in the bottom-left corner of Figure 5).

For a given model *m* (m=1,2,…,M), we process all queries from left to right in Figure 5. For each query $q_{n}$, the model retrieves its Rank-1 image from the gallery. If the identity (ID) of the retrieved Rank-1 image is consistent with the ground truth ID of $q_{n}$, the result is marked as a correct match and is not used in subsequent refinement. If the IDs differ, the retrieved image is marked as a Rank-1 error for that query, as indicated by the “If ID mismatched ⇒ mark as Rank-1 error” boxes in the middle row of the figure. Formally, for model *m* we obtain a set of Rank-1 error pairs $\left\{\left(q_{n},I_{n}^{\left(m\right)}\right)\right\}$, where $I_{n}^{\left(m\right)}$ denotes the top one mismatched image returned for query $q_{n}$.

In the next step (bottom row of Figure 5), we group these Rank-1 errors by query text. That is, for model *m* we aggregate, for each query $q_{k}$, its ground truth image–text pair $\left(q_{k}^{1},I_{k}^{1}\right)$ together with the corresponding mismatched Rank-1 image–text pair $\left(q_{k}^{2},I_{k}^{2}\right)$, forming an error group for that query. Each error group therefore approximates a situation where a short caption $q_{k}^{1}$ can plausibly describe both the ground truth image $I_{k}^{1}$ and another visually similar person image $I_{k}^{2}$ with a different ID.

Finally, as shown in the bottom box of Figure 5, we collect all error groups from the *M* TBPR models and obtain ${\left\{\left(\left(q_{k}^{1},I_{k}^{1}\right),\left(q_{k}^{2},I_{k}^{2}\right)\right)\right\}}_{k=1}^{K}$, where *K* is the total number of error groups aggregated over all models. These collected error groups serve as the input to the caption refinement stage described in Section 3.2, where multimodal large language models are used to expand and disambiguate the textual descriptions. Although the candidate set in Step 1 is obtained via retrieval models, we do not interpret the retrieved Rank-1 image as a semantically correct match, nor do we treat the query caption as objectively “incorrect”. Instead, the Rank-1 cross-ID result provides a visually similar comparison target (a hard negative) that helps expose which identity-specific cues are missing from the original caption. Importantly, we use multiple strong TBPR models with different backbones and training objectives, which often return different confusing negatives for the same query in practice. This makes the collected error groups complementary rather than tied to a single model’s failure mode, and it provides richer cross-image evidence for the subsequent MLLM-based caption expansion.

### 3.2. Step 2: MLLM-Based Caption Refinement

To refine the annotations of image–text pairs that are prone to annotation-induced mismatches, we leverage multimodal large language models so that multiple images and their associated textual descriptions can be considered simultaneously. This step corresponds to Step 2 in Figure 3. Step 2 is deliberately decoupled from the TBPR models used in Step 1. Once an error group is collected, the caption refinement is generated by Qwen-VL by jointly inspecting the two images and appending objective, identity-specific visual differences under a constrained prompt. In addition, we always preserve the original human-written sentence and only append expansions, which retains a stable semantic anchor and reduces the risk that the refinement becomes model-driven rewriting.

#### 3.2.1. Selection of the Multimodal Large Language Model

To conduct the annotation refinement, it is desirable to choose an MLLM that can provide reasonably detailed and fine-grained captions for person images. More importantly, our refinement in Step 2 requires the MLLM to jointly inspect multiple visually similar pedestrian images within a single prompt and to follow constrained instructions that append objective, identity-specific details for disambiguation. We therefore consider representative and publicly available MLLMs that can take images as input and follow instructions, while differing in capability/design so that we can assess their suitability for cross-image comparison and fine-grained description generation. We therefore conduct a qualitative comparison of three MLLMs—IDEFICS [31], LLaVA v1.6 [28] and Qwen-VL [29]—using person images as test data. In particular, IDEFICS is designed for interleaved image–text prompting and multi-image in-context reasoning, which aligns well with our cross-image comparison setting; LLaVA v1.6 represents a widely used instruction-tuned design that couples a CLIP-style vision encoder with a LLaMA-family language model and serves as a strong baseline for generating detailed captions, and Qwen-VL is designed for versatile vision–language understanding with grounding and text-reading capabilities, which can help capture fine-grained cues such as small accessories, logos, and carried objects that are useful for improving caption discriminability in TBPR. Specifically, we instruct each model to annotate pairs of similar images using the following prompt:

*“Assume you are working in the field of TBPR. You need to annotate the following two images in English. Please pay attention to the differences between the two images and explicitly include these differences in your annotations. Use the following format for your response: img1: ’caption1’; img2: ’caption2’.”*

**Figure 6:**
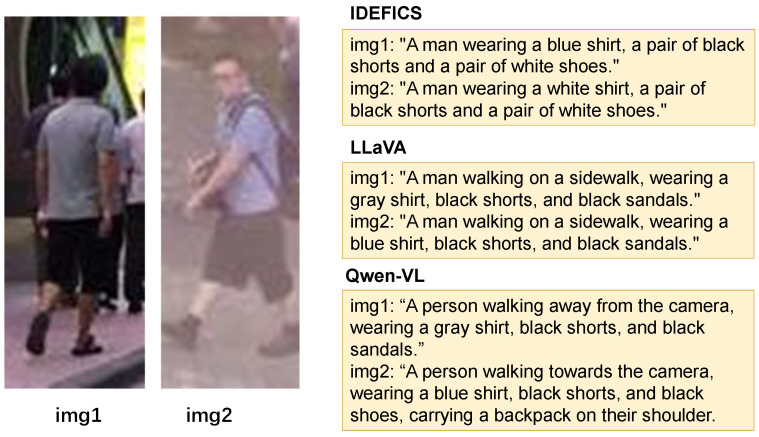
Comparison of the fine-grained image understanding performance of different MLLMs. In our qualitative tests on person images, Qwen-VL tends to capture more discriminative details and introduces fewer apparent hallucinations, so it is adopted in our refinement framework.

Through qualitative analysis, as shown in Figure 6, Qwen-VL appears to distinguish differences between images relatively well and exhibits fewer obvious hallucinations under our setting. Therefore, we adopt Qwen-VL as the MLLM for the subsequent annotation refinement step.

In addition to the qualitative comparison in Figure 6, we further conduct a quantitative comparison of IDEFICS, LLaVA v1.6 and Qwen-VL on CUHK-PEDES using the rewriting strategy in Section 4.6. We apply rewriting on the same IRRA-collected Rank-1 error groups and evaluate IRRA on the rewritten captions. As reported in Section 4.6, Qwen-VL achieves the best Rank@1 and mAP among the compared MLLMs, which is consistent with the qualitative observation and motivates our choice of Qwen-VL for caption refinement.

#### 3.2.2. Separate Expansion Followed by Merging

After the Rank-1 error pairs have been collected in Step 1 (Figure 5), we refine the captions associated with the ground truth image–text pairs by leveraging an MLLM. For every error pair, the ground truth pair $\left(q_{k}^{1},I_{k}^{1}\right)$ and its mismatched pair $\left(q_{k}^{2},I_{k}^{2}\right)$ are jointly fed into the MLLM so that the model can directly compare visually similar person and generate more discriminative textual descriptions.

For the refinement, we adopt a unified prompt to guide the expansion process. More specifically, we adopt a constrained prompting strategy for caption expansion rather than free-form generation or full rewriting. The prompt is designed to (i) operate on an error pair with two images (img1 and img2) so that the MLLM must compare them and explicitly mention visual differences, (ii) expand by appending additional content to the end of the original sentences (without rewriting or deleting the original text), and (iii) follow a strict single-line output format with reserved tags to support reliable parsing and lightweight post-processing (CleanText). We choose this strategy over alternative prompting approaches such as open-ended captioning or direct rewriting because in our setting the original human-written descriptions provide a stable semantic anchor, while unconstrained outputs and full replacements are more sensitive to omissions or hallucinations and are harder to standardize for large-scale refinement. The explicit formatting constraints also reduce post-processing ambiguity and make the refinement pipeline efficient and reproducible when applied to many error pairs. Concretely, for each error pair we send the ground truth image–text pair (img1) and its corresponding Rank-1 mismatched pair (img2) to the MLLM with the following instruction:

*You are working on text-based person retrieval. Your task is to expand the existing textual descriptions for two pedestrian images so that each description becomes more detailed and more discriminative. Image img1 corresponds to the following original description:* *<QUERY_TEXT>* *. Image img2 corresponds to the following original description:* *<GALLERY_TEXT>* *. Please carefully compare the two pedestrians and explicitly mention visual differences between them when expanding the captions (for example, clothes, colors, patterns, accessories, carried objects, hairstyle, posture, or surrounding context). The expansions must be written in English and should be appended to the end of the original sentences; do not rewrite or delete the original text. For easier post-processing, output only the additional content (the expansion) for each caption, without repeating the original sentences. Do not insert line breaks, bullet points, or quotation marks. Avoid emojis and special characters such as* *\n* *. Use the following format in a single line (no extra text before or after it):* *<EXP1_START> expansion for caption 1 <EXP1_END>; <EXP2_START> expansion for caption 2 <EXP2_END>* *. Do not output anything else. The expansions should be grammatically correct, concise, and avoid meaningless or overly subjective comments.*

After receiving the raw expansion from the MLLM, we apply a lightweight text cleaning function, denoted as CleanText. This function removes leading and trailing spaces, duplicated spaces, and trailing commas or semicolons; deletes line breaks, tab characters, and other control characters; strips quotation marks and unused marker symbols other than the reserved tags <EXP1_START>, <EXP1_END>, <EXP2_START>, and <EXP2_END>; normalizes repeated punctuation (for example, “!!” or “??”) to a single character; and discards purely empty or obviously meaningless expansions, such as outputs that contain only stopwords.

The MLLM outputs two expansions in the constrained format <EXP1_START> expansion for caption 1 <EXP1_END>; <EXP2_START> expansion for caption 2 <EXP2_END>. We parse this string, keep only the expansion for caption 1 (img1), i.e., the substring between <EXP1_START> and <EXP1_END>, clean it using CleanText, and then append the resulting segment to the current refined caption of the corresponding query. Each TBPR model is always evaluated on the same original test set, and error pairs from different models are processed separately. If a query appears in the error lists of multiple TBPR models, we call the MLLM multiple times (once per error pair) and sequentially append all cleaned expansions to the same caption. In this way, human-written descriptions are enriched with complementary, identity-specific details, while the iterative re-evaluation of TBPR models is avoided.

The overall procedure of this refinement step is summarized in Algorithm 1, where we iterate over all TBPR models and their collected error pairs, instantiate the prompt with $\left(q_{k}^{1},I_{k}^{1}\right)$ and $\left(q_{k}^{2},I_{k}^{2}\right)$, query the MLLM, extract and clean the expansion associated with img1, append it to the running version of the refined caption $q_{k,\mathrm{new}}^{1}$ if it is non-empty, and finally construct the refined test set $D^{\prime }$ by replacing each original caption $q_{j}$ with its expanded counterpart $q_{j}^{\mathrm{new}}$.
**Algorithm 1** Step 2: MLLM-Based Caption Expansion and Merging**Require:** Original test set D with query texts $\left\{q_{j}\right\}$ and images.**Require:** Error pairs ${\left\{E^{\left(m\right)}\right\}}_{m=1}^{M}$ from Step 1, where each $E^{\left(m\right)}={\left\{\left(\left(q_{k}^{1},I_{k}^{1}\right),\left(q_{k}^{2},I_{k}^{2}\right)\right)\right\}}_{k=1}^{N_{i}^{\left(m\right)}}$.**Require:** Multimodal LLM M (for example, Qwen-VL).**Require:** Prompt template described above.**Ensure:** Refined test set $D^{\prime }$ with expanded captions $\left\{q_{j}^{\mathrm{new}}\right\}$.  1:**for** m=1 to *M* **do**  2:    **for** k=1 to $N_{i}^{\left(m\right)}$ **do**  3:       Extract the ground truth pair $\left(q_{k}^{1},I_{k}^{1}\right)$ and the mismatched pair $\left(q_{k}^{2},I_{k}^{2}\right)$ from $E^{\left(m\right)}$.  4:       Instantiate the prompt $P_{k}^{\left(m\right)}$ by replacing <QUERY_TEXT> with $q_{k}^{1}$ and <GALLERY_TEXT> with $q_{k}^{2}$.  5:       Query the MLLM with images $I_{k}^{1}$ and $I_{k}^{2}$ and prompt $P_{k}^{\left(m\right)}$ to obtain the raw output string ${\mathrm{raw}\_\mathrm{out}}_{k}^{\left(m\right)}$.  6:       Parse ${\mathrm{raw}\_\mathrm{out}}_{k}^{\left(m\right)}$ using the marker tokens <EXP1_START> and <EXP1_END> to obtain the expansion for caption 1 (img1), denoted as $\Delta q_{k}^{1}$.  7:       Apply text cleaning to obtain $\hat{\Delta q_{k}^{1}}=$ CleanText $\left(\Delta q_{k}^{1}\right)$.  8:       **if** $\hat{\Delta q_{k}^{1}}$ is non-empty **then**  9:            Append the cleaned expansion to the current refined caption of the corresponding query:10:                   $q_{k,\mathrm{new}}^{1}\leftarrow q_{k,\mathrm{new}}^{1}+\hat{\Delta q_{k}^{1}}$.11:       **end if**12:    **end for**13:**end for**14:Construct the refined test set $D^{\prime }$ by replacing each original caption $q_{j}$ with its final expanded version $q_{j}^{\mathrm{new}}$.

### 3.3. Step 3: Discriminative Capability Evaluation

In addition to standard retrieval metrics such as Rank@K and mean Average Precision (mAP), we would like to evaluate whether the refined descriptions are intrinsically more discriminative, without relying on any specific TBPR model trained on the dataset. To this end, we introduce a set of metrics based on a frozen, pre-trained vision–language model (e.g., CLIP or SigLIP). This model is strictly used for evaluation and is not involved in the refinement process.

Given a test set with a gallery of person images ${\left\{I_{i}\right\}}_{i=1}^{N_{I}}$ and a set of query texts ${\left\{t_{j}\right\}}_{j=1}^{N_{T}}$, we keep a pre-trained vision–language model fully frozen and compute all metrics from its image–text similarity scores in three steps. First, we extract an image feature and a text feature using the frozen encoders $f_{I}$ and $f_{T}$, and then apply $\ell_{2}$ normalization so that cosine similarity can be computed by an inner product. Second, we compute the full cosine similarity matrix {s(i,j)} between all gallery images and all query texts and use it to obtain a similarity-based retrieval ranking for every query. Finally, based on these similarities and rankings, we compute the MeanMargin, AmbiguousRate(τ), and zero-shot CLIP_R@K as defined below. Since the vision–language model is frozen and never fine-tuned on the refined annotations, these metrics quantify the discriminability and ambiguity of the captions themselves. This is directly relevant to the annotation-induced mismatch problem studied in this paper: if one caption can semantically match multiple identities, the hardest cross-identity similarity becomes large, which reduces the margin and increases the ambiguous rate, and it also makes it less likely that the correct identity appears in the top-*K* results under a general-purpose vision–language embedding space.

We extract normalized features using the frozen CLIP image encoder $f_{I}$ and text encoder $f_{T}$:

(1) $$z_{i}^{I}=f_{I}\left(I_{i}\right),\;z_{j}^{T}=f_{T}\left(t_{j}\right).$$

The cosine similarity is defined as

(2) $$s\left(i,j\right)=\langle z_{i}^{I},z_{j}^{T}\rangle .$$

For clarity, the cosine similarity in its standard form is $s\left(i,j\right)=\langle z_{i}^{I},z_{j}^{T}\rangle /\left(∥z_{i}^{I}{∥}_{2}\;{∥z_{j}^{T}∥}_{2}\right)$. After $\ell_{2}$-normalization, ${∥z_{i}^{I}∥}_{2}={∥z_{j}^{T}∥}_{2}=1$, and so the cosine similarity reduces to the scalar product shown in Equation (2).

In practice, we $\ell_{2}$-normalize the resulting image and text features before computing s(i,j), so that $\langle z_{i}^{I},z_{j}^{T}\rangle$ is exactly the cosine similarity. For each query text $t_{j}$, we compute s(i,j) for all gallery images $I_{i}$ and sort the images in descending order of similarity, which yields a zero-shot retrieval ranking produced by the frozen vision–language model.

#### 3.3.1. Mean Discriminative Margin (MeanMargin)

For each query text $t_{j}$, let $I_{g(j)}$ denote its corresponding ground truth image in the gallery, where g(j) is an index mapping provided by the benchmark annotation. Let id(I) denote the identity (ID) label of an image *I*. We define the positive similarity as

(3) $$s_{\mathrm{pos}}\left(j\right)=s\left(g\left(j\right),j\right),$$

and the maximum negative similarity among all images with different identities as

(4) $$s_{\mathrm{neg}\_\max}\left(j\right)=\max_{i:\;\mathrm{id}(i)\neq \mathrm{id}(g(j))}s\left(i,j\right).$$

The discriminative margin for $t_{j}$ is then

(5) $$\mathrm{margin}\left(j\right)=s_{\mathrm{pos}}\left(j\right)-s_{\mathrm{neg}\_\max}\left(j\right).$$

The MeanMargin is the average over all descriptions:

(6) $$\mathrm{MeanMargin}=E_{j}\left[\mathrm{margin}\left(j\right)\right].$$

A higher MeanMargin suggests that, in the embedding space of the frozen vision–language model, the descriptions tend to be more aligned with their specific visual targets than with other identities. Here $s_{\mathrm{neg}\_\max}\left(j\right)$ corresponds to the hardest negative (the most similar gallery image with a different ID) under the frozen vision–language model. Therefore, a larger margin(j) indicates stronger separation between the ground truth match and the most confusing cross-identity match for the same text query, which is exactly what we expect to improve when annotation-induced mismatches are mitigated.

#### 3.3.2. AmbiguousRate(τ)

This metric measures the proportion of descriptions whose discriminability margin is small. For each description *j*, we compute its margin value margin(j) as in (5). Given a threshold τ (e.g., τ=0.01), we count how many descriptions have margin(j)<τ, and normalize by the total number of descriptions *N*:

(7) $$\mathrm{AmbiguousRate}\left(\tau \right)=\frac{1}{N}\left|\left\{j|\mathrm{margin}(j)<\tau \right\}\right|.$$

In other words, the AmbiguousRate(τ) is the fraction of descriptions whose margin falls below τ. A lower AmbiguousRate indicates fewer ambiguous descriptions in the dataset. In our experiments, we mainly report the cases τ=0 and τ=0.01, corresponding to ${\mathrm{Rate}}_{\mathrm{margin}<0}$ and ${\mathrm{Rate}}_{\mathrm{margin}<0.01}$, respectively.

Here, τ is applied to the margin values computed from cosine similarities. Intuitively, ${\mathrm{Rate}}_{\mathrm{margin}<0}$ measures how often the hardest negative image is even more similar than the ground truth image for the same text, while ${\mathrm{Rate}}_{\mathrm{margin}<0.01}$ measures how often the ground truth is not clearly separated from the hardest negative by a small margin. We emphasize that the threshold τ is only used to report the diagnostic metric AmbiguousRate(τ), and it is not used in Step 1 (Rank-1 error collection and grouping) or Step 2 (MLLM-based caption refinement). Therefore, the refinement framework itself does not depend on tuning τ, and changing τ only changes how we summarize the same margin distribution. We originally reported τ=0 and τ=0.01 because they correspond to two practically meaningful ambiguity levels: τ=0 counts cases where the hardest cross-identity negative is even more similar than the ground truth match (i.e., margin(j)<0), while τ=0.01 counts near-ambiguous cases where the ground truth is not clearly separated from the hardest negative by a small margin. By definition, the AmbiguousRate(τ) is the fraction of samples whose margins fall below τ, and is thus monotonically non-decreasing with respect to τ. To further demonstrate that our conclusion about reduced ambiguity is not sensitive to selecting a particular τ, we provide a sensitivity analysis that scans τ in [−0.05,0.05] with a step size of 0.005 under frozen ViT-bigG-14 (CLIP) and SigLIP-large in the ablation study (Section 4.6).

#### 3.3.3. Zero-Shot CLIP_R@K

We also perform zero-shot retrieval using the frozen vision–language model. For each textual description, we rank all images according to the similarity s(i,j) and compute Recall@K in the usual way, which we denote as CLIP_R@K. An improvement in CLIP_R@K directly reflects that the refined text contains more identity-specific visual cues that are recognizable by a general vision–language model. In the experimental section, we instantiate this step with ViT-bigG-14 (CLIP) and SigLIP-large.

Concretely, for each query text $t_{j}$ with the paired ground truth image $I_{g(j)}$, we obtain a ranking of all gallery images by sorting s(i,j) in descending order and take the top-*K* results. We then check whether the top-*K* ranked images contain at least one image $I_{i}$ whose identity equals that of the ground truth image, i.e., $\mathrm{id}\left(I_{i}\right)=\mathrm{id}\left(I_{g(j)}\right)$. We average this indicator over all query texts to obtain CLIP_R@K (reported as a percentage in our tables). This definition aligns with the standard Rank@K (Recall@K) in TBPR, but uses the frozen vision–language model similarity s(i,j) to produce the ranking, so it reflects dataset-level caption discriminability.

## 4. Experiments

In this section, we conduct extensive experiments on TBPR benchmarks and provide a detailed analysis to verify the effectiveness of our proposed methods.

### 4.1. Datasets and Metrics

(1) Datasets: We conducted extensive experiments primarily on the CUHK-PEDES dataset [1], which remains the most widely used and representative benchmark for TBPR. It is notable for its diversity and large scale, which comprises 40,206 images and 80,440 textual descriptions, corresponding to 13,003 unique person identities. The images are sourced from five established person re-identification datasets—VIPer, CUHK01, CUHK03, Market-1501, and SSM—captured by multiple surveillance cameras spanning various time periods, locations, and viewpoints, thereby ensuring the robustness and diversity of the dataset. Each image in CUHK-PEDES is paired with two textual descriptions, with an average sentence length of 23 words. These descriptions encompass information regarding appearance, actions, poses, and interactions with surrounding objects.

To further validate the generalizability and effectiveness of our proposed approach, we additionally employed two large-scale TBPR datasets: ICFG-PEDES [4] and RSTPReid [3].

ICFG-PEDES contains 54,522 images corresponding to 4102 identities. Each image is annotated with a single textual description. The dataset is partitioned into a training set and a test set: the training set comprises 34,674 image–text pairs from 3102 identities, while the test set consists of 19,848 image–text pairs from the remaining 1000 identities.

RSTPReid consists of 20,505 images of 4101 identities, acquired from 15 different cameras. Each identity has five images captured by different cameras, and each image is annotated with two textual descriptions. Following the official data split, the training, validation, and test sets contain 3701, 200, and 200 identities, respectively.

(2) Metrics: To comprehensively evaluate retrieval performance, we employ two widely used metrics: Rank@K (K = 1, 5, 10) and mean Average Precision (mAP). Higher values for these metrics indicate better retrieval performance.

Rank@K, also referred to as Recall@K, measures the proportion of queries for which at least one relevant image is retrieved within the top K results. Suppose there are *N* queries; for each query $q_{i}$, let $S_{i}$ denote the set of retrieved images ranked by similarity to $q_{i}$, and $G_{i}$ denote the set of ground truth images corresponding to $q_{i}$. The indicator function $I_{i}^{K}$ is defined as follows:   

(8) $$I_{i}^{K}=\left\{\begin{array}{cc} 1, & \mathrm{if}\;G_{i}\cap \mathrm{TopK}\left(S_{i}\right)\neq \emptyset \\ 0, & \mathrm{otherwise} \end{array}\right.$$

where $\mathrm{TopK}(S_{i})$ denotes the top *K* retrieved images for query $q_{i}$. The Rank@K is then calculated as 

(9) $$\mathrm{Rank}@K=\frac{1}{N}\sum_{i=1}^{N}I_{i}^{K}$$

where *N* is the total number of queries.

The mean Average Precision (mAP) is adopted to evaluate the overall ranking quality of the retrieval results. For each query $q_{i}$, let $n_{i}$ be the number of relevant images, and let $r_{i,1},r_{i,2},\ldots ,r_{i,n_{i}}$ denote the ranks at which the relevant images are retrieved. The average precision (AP) for $q_{i}$ is defined as

(10) $${\mathrm{AP}}_{i}=\frac{1}{n_{i}}\sum_{k=1}^{n_{i}}P_{i}\left(r_{i,k}\right)$$

where $P_{i}\left(r_{i,k}\right)$ denotes the precision at the $r_{i,k}$-th position for query *i*. The mean Average Precision (mAP) is then computed as the mean of AP over all queries:

(11) $$\mathrm{mAP}=\frac{1}{N}\sum_{i=1}^{N}{\mathrm{AP}}_{i}$$

where *N* is the number of queries.

In addition to Rank@K and mAP, we also report the discriminative capability metrics defined in Section 3.3, including the Mean Discriminative Margin (MeanMargin), AmbiguousRate(τ), and zero-shot CLIP_R@K.

### 4.2. Implementation Details

In our experiments with trainable TBPR models, we selected four state-of-the-art models: RaSa [22], RDE [23], TBPS-CLIP* [20] and IRRA [14]. We choose these four baselines because they are representative state-of-the-art methods with publicly available codebases and they are representative of several mainstream directions in current TBPR research. In particular, TBPS-CLIP* represents CLIP-based TBPR baselines that directly leverage large-scale vision–language pretraining and have become strong and commonly used references, IRRA represents approaches that enhance fine-grained cross-modal alignment by implicit relation reasoning and aligning, RaSa represents relation and sensitivity aware representation learning with an emphasis on hard negative discrimination and robustness, and RDE represents noisy correspondence learning that explicitly addresses imperfect text–image correspondences. Taken together, these models cover CLIP-style pre-training, relation modeling for image–text matching, and robustness under noisy supervision, and therefore provide a representative view of the current TBPR research landscape. Here, TBPS-CLIP* denotes a simplified, lightweight variant of TBPS-CLIP that we used throughout our experiments; we adopted the simplified version of TBPS-CLIP to ensure its parameter count was comparable to the other models. Since the official model weights were not publicly released, we reproduced all results using the authors’ publicly available codebases. All evaluations were conducted on two NVIDIA 3080 GPUs. To further examine whether ASM can also benefit recent fine-grained TBPR methods and to provide evidence beyond the models used in Step 1, we additionally reproduce and evaluate FCSA [15] using the authors’ public implementation.

For clarity, all TBPR models used in this work were reproduced based on publicly available implementations released by the corresponding authors, rather than by directly using the reported numbers from previous papers. This is also because our ASM evaluation uses a refined version of the test captions (i.e., a new test annotation file), so the results reported in prior papers on the original test captions cannot be directly reused for fair comparison. We used the official data splits and evaluation protocols of CUHK-PEDES, RSTPReid and ICFG-PEDES, and we followed the experimental settings provided in the released implementations to ensure fair comparison. When comparing the “Original” and “ASM”, for each method we kept the model and its experimental settings unchanged and only replaced the dataset annotation files with the refined captions for evaluation.

**Figure 7:**
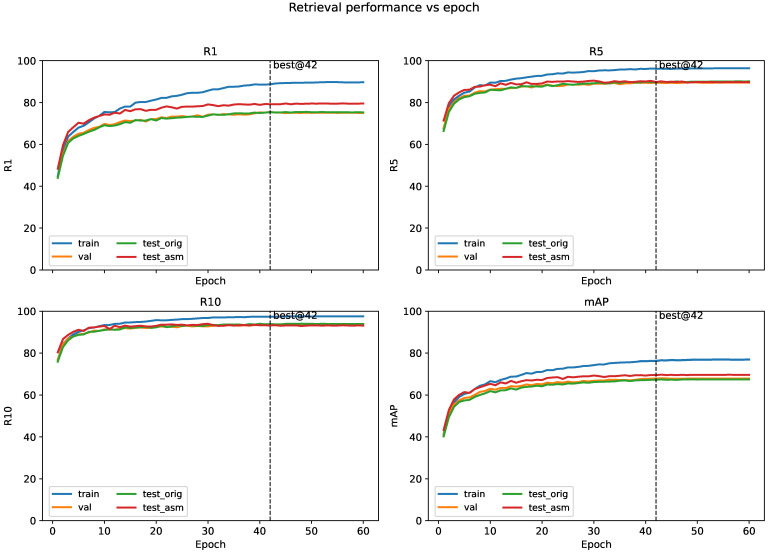
Retrieval performance versus epoch on CUHK-PEDES. We plot Rank@1, Rank@5, Rank@10 and mAP on train, val, test_orig (original captions) and test_asm (refined captions). The black dotted vertical line marks the epoch of the best validation Rank@1 checkpoint.

To better characterize the training quality and the evolution of retrieval performance across training, validation and testing stages, we additionally recorded per-epoch retrieval curves during model training. Figure 7 reports Rank@1, Rank@5, Rank@10 and the mAP versus epoch on the training split, the validation split, the original test split, and the refined (ASM) test split. Since the training split and the test split are relatively large, evaluating the full splits at every epoch is time consuming; therefore, for per-epoch monitoring we evaluated the validation split using the full validation set, and evaluated the training split and both test variants using a fixed, randomly sampled subset whose identity scale matched that of the validation split. The subset was sampled once with a fixed random seed and kept unchanged across epochs, ensuring fair and reproducible comparisons. For CUHK-PEDES, our refined annotation file modified only the test captions while keeping the image paths and identity labels unchanged; thus, the performance gap between test_orig and test_asm curves isolated the effect of caption refinement under the same image gallery. We also emphasize that the best checkpoint is selected based on validation Rank@1 only, and the test curves are reported for diagnostic visualization rather than model selection.

### 4.3. Zero-Shot Evaluation with Frozen Vision–Language Models

To assess whether the refined descriptions are intrinsically more discriminative, we first perform a zero-shot evaluation using frozen vision–language models on the modified portion of the CUHK-PEDES test set. Specifically, we adopt ViT-bigG-14 (CLIP) and SigLIP-large as the image–text encoders and compute CLIP_R@K, MeanMargin, and the proportion of ambiguous descriptions, i.e., Rate_margin <0 and Rate_margin <0.01, which correspond to AmbiguousRate with thresholds τ=0 and τ=0.01, respectively.

For each encoder, we follow the evaluation procedure described in Section 3.3: we extract $\ell_{2}$-normalized image features for all gallery images and $\ell_{2}$-normalized text features for all query descriptions using the frozen encoders, compute cosine similarities for all image–text pairs, and then compute CLIP_R@K, MeanMargin, and Rate_margin <0/Rate_margin <0.01 from these similarity scores. To avoid any influence from model training, the encoders are never fine-tuned on our refined annotations; the evaluation is purely based on feature extraction and similarity ranking under a fixed, pre-trained vision–language model.

The models are kept frozen and are not fine-tuned on our refined annotations. Thus, any performance difference reflects changes in the quality and discriminability of the test captions rather than model adaptation.

From Table 2, we observe similar trends for both encoders. Zero-shot CLIP_R@K becomes higher after refinement, MeanMargin moves towards a larger value, and the fractions of ambiguous descriptions (Rate_margin <0 and Rate_margin <0.01) become lower. Since the models are frozen, these differences suggest that our Separate Expansion Followed by Merging strategy makes the benchmark captions more discriminative and better aligned with their corresponding images. More concretely, for ViT-bigG-14 (CLIP), zero-shot CLIP_R@1, CLIP_R@5, and CLIP_R@10 increase by 39.7%, 23.3%, and 20.7% respectively after refinement, while the MeanMargin improves by 17.9%. At the same time, the proportions of descriptions whose margins fall below 0 and 0.01 (Rate_margin <0 and Rate_margin <0.01) are reduced by 6.1% and 4.7% respectively. A similar pattern is observed for SigLIP-large, where CLIP_R@1, CLIP_R@5, and CLIP_R@10 improve by 53.4%, 36.1%, and 32.3%, and the MeanMargin increases by 50.7%. The ambiguous description rates are substantially reduced by 17.0% (Rate_margin <0) and 12.2% (Rate_margin <0.01). Since both encoders are frozen and never see the refined annotations during training, these consistent gains strongly suggest that our Separate Expansion Followed by Merging strategy indeed enhances the intrinsic discriminability of the captions, rather than overfitting to any particular TBPR model. In other words, the refined texts provide clearer, identity-specific cues that are more easily distinguishable from other identities in the joint image–text embedding space.

To further analyze the experimental results in multiple dimensions, we provide a more detailed analysis of Table 2. First, the improvements are consistent across both ViT-bigG-14 (CLIP) and SigLIP-large: after refinement, CLIP_R@K increases, MeanMargin becomes larger, and Rate_margin <0/Rate_margin <0.01 decrease, which indicates that the observed gains are not tied to a particular encoder choice and are instead attributable to the refined captions being more discriminative. Second, across different *K* values, the absolute gains of CLIP_R@K are maintained from Rank@1 to Rank@10 for both encoders, while the relative change is larger at smaller *K* (e.g., CLIP_R@1) and becomes smaller as *K* increases. This trend suggests that refinement mainly strengthens the most relevant identity-specific cues that affect the top-ranked results, while the benefit naturally saturates when more candidates are included in the top-*K* list. Third, the changes in the MeanMargin and ambiguity rates provide complementary evidence beyond CLIP_R@K: although the MeanMargin remains negative before and after refinement, it consistently moves towards a larger value, meaning that the hardest cross-identity negative similarity is still strong in this challenging setting but becomes less dominant after refinement; meanwhile, the decreases of Rate_margin <0 and Rate_margin <0.01 indicate that fewer descriptions fall into highly ambiguous cases where the hardest negative is comparable to or even exceeds the ground truth match. Taken together, these multi-dimensional observations support the same conclusion: the refined captions reduce annotation-induced mismatches by improving caption discriminability and by increasing the separation between the ground truth identity and confusing cross-identity matches under a frozen vision–language embedding space.

### 4.4. Human Evaluation of Caption Quality

The main experiments in Section 4.3 quantify the benefit of refined annotations through model-based evaluations (frozen vision–language diagnostics and mainstream TBPR performance). However, improved benchmark reliability should also be reflected in how humans perceive the refined captions in terms of language quality and image-grounded usefulness. We therefore conduct a small-scale human evaluation on CUHK-PEDES to directly assess whether refined captions are more natural and more useful/faithful from a human perspective.

We randomly sample N=300 query instances from the modified portion of the CUHK-PEDES test set (the same evaluation subset used in Table 2). For each instance, we show the evaluator two images: IMG1 is the query (ground truth) person image, and IMG2 is a visually similar gallery image that appears as a rank-1 mismatch (a typical confusing distractor identity) in the error groups collected in Step 1. We then show two candidate captions for IMG1: the original human-written caption and the refined caption produced by our Separate Expansion Followed by Merging strategy. To reduce presentation bias, the two captions are anonymized and randomly permuted as A/B for each instance.

The evaluator assigns two scores in [0,10] to each caption (0 = very poor; 10 = excellent). Naturalness measures the fluency and grammatical correctness of the text. Useful&Faithful measures whether the caption is faithful to IMG1 and simultaneously helpful for distinguishing IMG1 from IMG2 by explicitly describing identity-specific visual cues, while hallucinated or unsupported details are penalized. In addition to the numeric scores, the evaluator provides a paired label (A, B, tie, or both_bad) indicating which caption is better under each criterion. For concise summarization, we further report Final, computed as the arithmetic mean of Naturalness and Useful&Faithful.

Table 3 reports descriptive statistics and Table 4 reports paired outcomes and statistical tests. Overall, refined captions achieve higher mean scores on both dimensions, with mean improvements of +1.02 in Naturalness and +1.09 in Useful&Faithful. The mean improvements are statistically significant under a two-sided exact sign test on per-instance score differences (Naturalness: $p=8.88\times 10^{-14}$; Useful&Faithful: $p=7.60\times 10^{-10}$), and the 95% confidence intervals for the mean improvements (both bootstrap and normal approximation) do not overlap zero. In the paired preference labels, excluding ties, refined captions win 73.44% of the time for Naturalness (Wilson 95% CI [67.53,78.62]%) and 68.35% for Useful&Faithful (Wilson 95% CI [62.66,73.53]%). These results provide direct human-facing evidence that the refined annotations are perceived as more natural and more image grounded/useful for distinguishing confusing identities, which complements the model-based improvements reported in the main experiments.

### 4.5. Results with Mainstream TBPR Models

In this section, we report how four representative TBPR models behave when evaluated on the original versus refined annotations. All the results of the compared methods in this subsection are obtained by our reproduction using publicly available implementations under the same official data splits and evaluation protocols, and we do not rely on the reported numbers from previous papers. These results serve as supporting evidence for the zero-shot findings in Table 2: although they are influenced by training details and model capacity, they confirm that better test annotations also translate into higher end-to-end retrieval performance.

As shown in Table 5, our approach resulted in the following Rank@1 accuracies on CUHK-PEDES: 81.17 for RaSa, 80.34 for RDE, 79.34 for TBPS-CLIP, and 79.37 for IRRA. The Rank@1 accuracies increased significantly after annotation refinement. Moreover, the proposed method achieves robust improvements across most metrics. For example, Rank@1 and mAP consistently improved across all models. This indicates that expanding captions separately before merging effectively captures the uniqueness of each caption, thereby reducing the number of different identities associated with the same caption.

Although ASM brings consistent gains in Rank@1 and mAP, we also observe a few non-improving higher-K recalls on CUHK-PEDES. In Table 5, RDE shows a slight Rank@10 drop (93.97 to 93.63; −0.34), and IRRA exhibits marginal decreases at Rank@5 (89.46 to 89.39; −0.07) and Rank@10 (93.81 to 92.58; −1.23), even though both methods still achieve substantial improvements on Rank@1 and mAP. We attribute these rare decreases at larger K mainly to a precision–recall trade-off introduced by making captions more discriminative: the appended expansions are generated by contrasting the query image against a confusing cross-ID hard negative, so they tend to emphasize fine-grained and sometimes image-specific cues. Such cues are effective for resolving the hardest confusing negatives and improving early-rank precision, but they can also reshuffle the long-tail ranking for a small fraction of queries, especially because the TBPR models are trained on the original (shorter) caption distribution and are evaluated on longer refined sentences with rarer attribute words. In addition, under challenging visual conditions (occlusion or low resolution), a small fraction of expansions may contain uncertain or weakly identity-related details, which can slightly affect Rank@5/10 while leaving the overall trend unchanged. These observations motivate additional safeguards discussed in Section 5, such as imposing a stricter expansion budget, discouraging background-only cues, and filtering low-confidence expansions using a frozen vision–language consistency check.

We further verify the effectiveness of our strategy on RSTPReid and ICFG-PEDES. The results for RSTPReid are reported in Table 6, while those for ICFG-PEDES are shown in Table 7. Consistent with the previous results on the CUHK-PEDES benchmark, the proposed strategy leads to substantial improvements in Rank@1 accuracy on both the RSTPReid and ICFG-PEDES datasets. These results further demonstrate the robustness and generalizability of our approach across different benchmarks, providing strong evidence for its effectiveness as an annotation refinement strategy.

It is worth noting that, unlike the frozen CLIP/SigLIP evaluation in Table 2, the performances of these TBPR models depend on specific training settings and model choices. They are therefore interpreted as complementary evidence: they show that once the test annotations become more discriminative, a variety of existing TBPR models can better exploit them and achieve higher end-to-end retrieval accuracy.

### 4.6. Ablation Study

In addition to the four representative TBPR models evaluated above, we further conduct an extra experiment with a recent advanced method, FCSA [15], to verify that the improvement brought by ASM is not limited to the specific models used in Step 1. Table 8 reports the “Original vs. ASM” results on CUHK-PEDES under the same evaluation protocol, where the FCSA model is kept unchanged and only the test captions are replaced by our refined annotations.

As shown in Table 8, ASM improves FCSA by +2.85 Rank@1 and +1.74 mAP, indicating that the refined captions can also benefit a recent fine-grained alignment method that is outside the Step 1 model set. We note that Rank@5/Rank@10 slightly decrease, which is consistent with the precision–recall trade-off observed in Table 5: ASM makes the descriptions more discriminative and often more query specific by emphasizing identity-specific differences against hard negatives, which tends to boost early-rank precision and overall ranking quality (mAP) while occasionally reshuffling the long-tail retrieval order for a small fraction of queries. Overall, this experiment serves as complementary evidence that our caption refinement is not tied to a particular TBPR architecture or the specific models used for mismatch discovery.

To further validate the design of our Separate Expansion Followed by Merging strategy, we compare it with two alternative expansion-based caption refinement strategies: Parallel Expansion and Serial Expansion.

Parallel Expansion: In this strategy, multiple sets of image–text pairs that might cause mismatches are merged in parallel and then their textual descriptions are expanded. For each of the *M* TBPR models, incorrect image–text pairs are collected and then merged. The MLLM generates expanded descriptions for each group simultaneously, which are then appended to the original dataset.

Serial Expansion: In this strategy, the texts in image–text pairs that are likely to cause mismatches are iteratively refined. The workflow proceeds iteratively on a per-model basis. For each model *m*, mismatches are identified and refined. The updated dataset is then used to evaluate the next model m+1, and so on.

We conducted an ablation study using the IRRA model [14] on the CUHK-PEDES dataset, as shown in Table 9. Compared with the original captions, both Parallel Expansion and Serial Expansion improve Rank@1 and mAP, while Separate Expansion Followed by Merging achieves the best performance.

To present the validation to confirm that the differences observed in Table 9 are not by chance, we further conduct statistical significance testing on per-query retrieval results over the same N=6156 queries. For Rank@K (a paired binary indicator for each query), we use the exact two-sided McNemar test. For mAP, we compute per-query Average Precision and report the paired *t*-test, together with paired bootstrap 95% confidence intervals (20,000 resamples) and a paired sign–flip permutation test (20,000 permutations). Table 10 reports the results.

Table 10 shows statistically significant improvements of Separate Expansion Followed by Merging over Parallel Expansion and Serial Expansion on Rank@1, Rank@5, Rank@10 and mAP (for example, compared with Parallel Expansion, Rank@1 increases by +4.191 percentage points with McNemar $p=8.75\times 10^{-21}$, and mAP increases by +2.404 percentage points with a 95% bootstrap confidence interval [1.919, 2.895] and paired *t*-test $p_{t}=7.09\times 10^{-22}$). Compared with the Original, Rank@1 increases by +5.669 percentage points with McNemar $p=5.85\times 10^{-36}$ and mAP improves by +2.804 percentage points with a 95% bootstrap confidence interval [2.324, 3.292] and paired *t*-test $p_{t}=7.17\times 10^{-30}$; the difference at Rank@5 is not statistically significant (p=0.9027), and Rank@10 changes slightly (Δ=−1.235 percentage points, $p=2.00\times 10^{-4}$), which is consistent with the refined captions being more query-specific and mainly benefiting early-rank retrieval and overall ranking quality.

**Figure 8:**
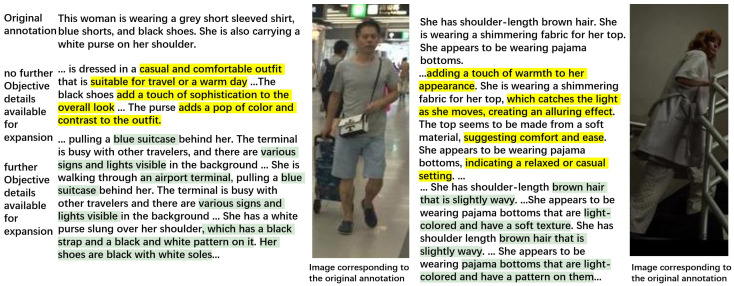
Visualization analysis of the reasons for the decline in Rank@5 and Rank@10 performance. Top: Under serial expansion, once objective details are exhausted, the model introduces subjective, low-information phrases (yellow highlights), which dilutes discriminative cues. Bottom: With separate expansion followed by merging, each round restarts from the original annotation and consistently reinforces objective evidence (green highlights). Yellow denotes subjective/low-information additions; green denotes objective details.

It is noted that while Serial Expansion improves Rank@1, it often leads to a decline in Rank@5 and Rank@10 performance. To understand this, as shown in the upper part of Figure 8 (yellow highlights), we must consider that once the observable details are exhausted during the iterative process, the MLLM is forced to keep expanding and starts producing subjective, low-information phrases such as “a casual and comfortable outfit”, “adds a touch of sophistication”, “adds a pop of color and contrast”, or “the fabric catches the light, creating an alluring effect”. These subjective additions dilute the discriminative cues that tie the caption to the target person image, causing the correct match to drop out of the top five.

In contrast, the Separate Expansion Followed by Merging strategy largely avoids the Rank@10 degradation observed in Serial Expansion. As shown in Table 9, Rank@5 stays essentially on par with the Original captions, and Rank@10 remains higher than Parallel Expansion and Serial Expansion, although it is slightly lower than the Original captions (Table 10). Because each round restarts from the original annotation, the MLLM tends to reinforce observable evidence rather than accumulating speculative content across iterations. As shown in the lower part of Figure 8 (green highlights), the MLLM consistently focuses on concrete details, for example, “pulling a blue suitcase”, “various signs and lights are visible”, “a white purse with a black strap and a black-and-white pattern”, and “black shoes with white soles”. While this may introduce some redundancy and make the refined captions more query specific, it avoids subjective or low-information additions, thereby preserving discriminative cues and mitigating the pronounced Rank@5/Rank@10 drops caused by iterative speculation in Serial Expansion.

To make our choice of MLLM more principled, we further conduct a quantitative comparison of IDEFICS, LLaVA v1.6, and Qwen-VL under the same rewriting setting on CUHK-PEDES with IRRA. Specifically, rewriting is applied to the Rank-1 error groups collected in Step 1; the MLLM is provided with the ground truth image $I_{k}^{1}$ together with the corresponding confusing image $I_{k}^{2}$, and is asked to rewrite $q_{k}^{1}$ into a more discriminative description for $I_{k}^{1}$. We then evaluate IRRA using the rewritten captions. As reported in Table 11, Qwen-VL achieves the best Rank@1 (63.32) and mAP (56.78), outperforming LLaVA v1.6 (Rank@1 60.80; mAP 55.60) and IDEFICS (Rank@1 57.89; mAP 53.83). Although LLaVA v1.6 attains slightly higher Rank@5/Rank@10, Qwen-VL provides the strongest improvements on the primary metrics we target (Rank@1 and mAP), which is consistent with our qualitative observations and motivates our choice of Qwen-VL for caption refinement.

**Figure 9:**
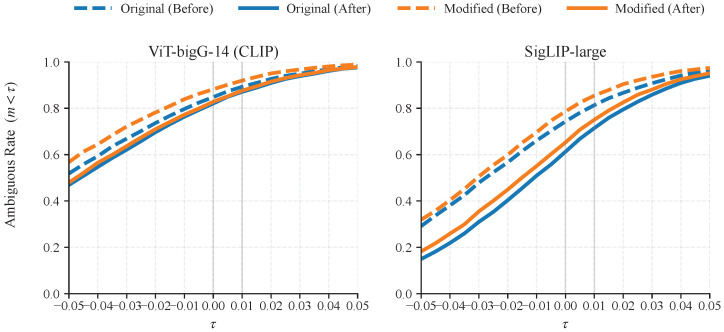
AmbiguousRate(τ) as a function of the threshold τ under frozen ViT-bigG-14 (CLIP) and SigLIP-large on CUHK-PEDES. “Original” denotes the full test set, and “Modified” denotes the modified portion used in Table 2. Dashed lines denote captions before refinement, and solid lines denote captions after refinement. The two vertical gray lines mark the two reference thresholds τ=0 and τ=0.01 reported in the main paper.

In Section 3.3, AmbiguousRate(τ) is introduced as a dataset-level diagnostic metric computed from the margin values defined by the frozen vision–language similarity scores. We conduct an additional sensitivity analysis; specifically, we scan τ from −0.05 to 0.05 with a step size of 0.005 and compute the AmbiguousRate(τ) under frozen ViT-bigG-14 (CLIP) and SigLIP-large on two evaluation sets: the full CUHK-PEDES test set (denoted as “Original”) and the modified portion used in Table 2 (denoted as “Modified”). Figure 9 plots the resulting curves. For both encoders and across the entire scanned range of τ, the refined captions consistently yield lower AmbiguousRate(τ) than the original captions on both the “Original” and “Modified” sets, which indicates that the observed reduction of ambiguity is not tied to a specific choice of τ. Table 12 lists the representative values on the “Modified” portion, including the two reference thresholds τ=0 and τ=0.01 reported in the main paper.

### 4.7. Qualitative Results Analysis

**Figure 10:**
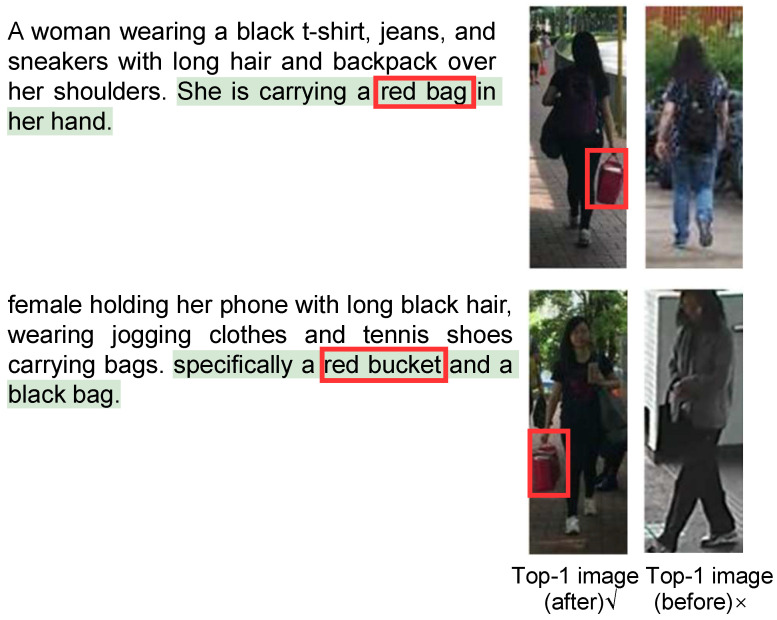
Visualization of example results on CUHK-PEDES. The green text highlights the expanded description generated by the MLLM, while the red box marks the key distinguishing feature between the two images as identified by the MLLM. These images show the results after the first iteration of Separate Expansion Followed by Merging. The TBPR model’s Rank-1 retrieval result is corrected from the previously incorrect image (**right**) to the correct image (**left**).

**Figure 11:**
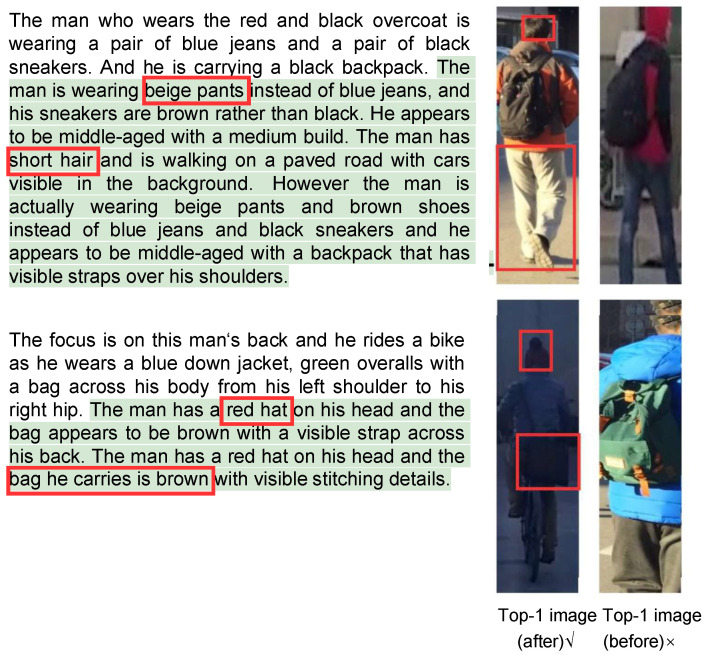
Visualization of example results on RSTPReid. The green text highlights the expanded description generated by the MLLM, while the red box marks the key distinguishing feature between the two images as identified by the MLLM. After applying Separate Expansion Followed by Merging, the TBPR model’s Rank-1 retrieval result is corrected from the previously incorrect image (**right**) to the correct image (**left**), demonstrating the effectiveness of the refined annotation.

**Figure 12:**
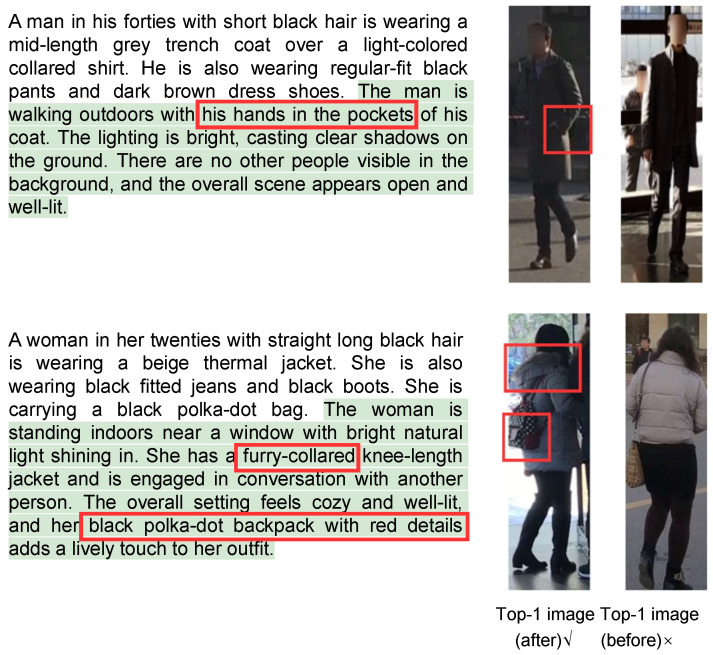
Visualization of example results on ICFG-PEDES. The green text highlights the expanded description generated by the MLLM, while the red box marks the key distinguishing feature between the two images as identified by the MLLM. After applying Separate Expansion Followed by Merging, the TBPR model’s Rank-1 retrieval result is corrected from the previously incorrect image (**right**) to the correct image (**left**), demonstrating the effectiveness of the refined annotation.

We visualized the examples of the retrieval results on all three benchmarks. As shown in Figure 10, the figure presents the captions before and after refinement, as well as the top one image retrieved by the TBPR model based on the original and refined captions. Specifically, the green text highlights the expanded description generated by the MLLM, and the red box marks the key distinguishing feature between the two images as identified by the MLLM. These images show the results after the first iteration of Separate Expansion Followed by Merging. It can be observed that the MLLM expanded the caption with more details, for example, “She is carrying a red bag in her hand”. This additional detail enables the TBPR model to distinguish the correct image (left) from the previously incorrect one (right). Therefore, these results demonstrate that our framework effectively reduces the cases where a single caption corresponds to multiple person IDs in the dataset and improves fine-grained discrimination ability. As shown in Figure 11 and Figure 12, we present the visualizations of the example captions before and after refinement, as well as the top retrieval results on the RSTPReid and ICFG-PEDES datasets. Similarly to the results on CUHK-PEDES, the refined captions generated by the MLLM contain more distinctive details, which help the TBPR model to better distinguish the correct image from similar ones. These results further demonstrate the robustness and generalizability of our method in reducing the instances where a single caption corresponds to multiple identities across different datasets. 

## 5. Discussion

Although ASM yields consistent improvements in caption discriminability and benchmark reliability, it still has practical limitations (e.g., the dependence on the adopted MLLM for fine-grained perception, the additional cost when scaling refinement, and the model-based candidate discovery in Step 1), which we discuss in this section together with possible remedies and future directions.

The refinement stage in Step 2 depends on the fine-grained image understanding ability of the adopted MLLM. Although we use a constrained prompt that appends expansions to the end of the original sentences and apply CleanText for lightweight post-processing, the generated expansions may still contain redundancy, uneven levels of detail across samples, or occasional uncertain statements in challenging cases (e.g., small accessories, occlusion, or low-resolution images). In addition, longer refined descriptions may increase text length and thus slightly increase inference cost in downstream retrieval. Possible improvements include further constraining the expansion length, removing repeated or low-information phrases after merging, and introducing an automatic consistency check that compares the refined text with the paired image using a frozen vision–language model similarity, so that expansions that reduce image–text consistency can be filtered or revised while preserving the stability brought by retaining the original human-written captions.

When the scaling MLLM calls to larger portions of a benchmark or to multiple benchmarks, the refinement cost can be non-trivial, because the framework queries the MLLM for many error pairs and performs separate expansions followed by merging. In our current setting, we focus on refining the test sets to improve benchmark reliability and evaluation fairness, but extending the same process to broader splits may require more careful efficiency design. A practical recommendation is to cache MLLM outputs for repeated pairs, batch refinement requests when supported by the MLLM service, and prioritize refinement by the severity of ambiguity (for example, by margin-based metrics such as MeanMargin and Rate_margin <0/Rate_margin <0.01 under a frozen vision–language model), so that computational resources are allocated to the most ambiguous descriptions first.

Finally, we acknowledge that the discovery stage in Step 1 is still influenced by the set of TBPR models used to collect Rank-1 errors: ambiguous cases that current models already handle well may be under-sampled, while some collected hard negatives may reflect genuine model weaknesses rather than annotation ambiguity. However, we mitigate this risk by aggregating multiple heterogeneous TBPR models and by validating the refined captions using frozen vision–language diagnostics and human evaluation, designing a more model-agnostic discovery strategy remains an important future direction. For example, ambiguity candidates may be identified by cross-identity duplicate or near-duplicate caption detection purely from text, by grouping visually similar pedestrians via unsupervised image clustering, or by screening low-margin queries under a frozen general-purpose vision–language model, followed by lightweight human verification before large-scale refinement.

## 6. Conclusions

In this paper, we addressed the annotation-induced mismatching problem in TBPR and proposed a novel annotation refinement framework to generate more detailed and distinctive textual descriptions by leveraging multimodal large language models (MLLMs). To the best of our knowledge, this is the first work to explicitly mitigate annotation-induced mismatches by refining textual annotations with multiple reference images simultaneously, in contrast to previous methods that only operate on a single image at a time. We introduced a Separate Expansion Followed by Merging strategy and a set of discriminative capability metrics based on frozen vision–language models, including zero-shot CLIP_R@K, MeanMargin, and ambiguous description rates (Rate_margin <0 and Rate_margin <0.01). On the modified portion of the CUHK-PEDES test set, frozen ViT-bigG-14 (CLIP) and SigLIP-large exhibit substantial zero-shot improvements (e.g., CLIP_R@1 gains of 39.7% and 53.4%, respectively), increased MeanMargin, and markedly reduced ambiguous rates, which together indicate that the refined captions are intrinsically more discriminative and better aligned with their ground truth images. Furthermore, comprehensive experiments with multiple TBPR models on three popular benchmarks (CUHK-PEDES, RSTPReid, and ICFG-PEDES) demonstrate that the refined annotations consistently boost Rank@1 and mAP, highlighting the practical benefits of our refinement framework for real-world TBPR tasks.

Future work will encompass exploring more powerful and efficient multimodal large language models that can provide finer-grained and more reliable descriptions under constrained computation budgets, for example by leveraging mixture-of-experts architectures, instruction tuning tailored to person-centric scenes, and tighter integration with CLIP-style vision encoders. Another promising direction is to unify refinement and retrieval learning in a joint or iterative optimization loop, so that retrieval models can actively query the refinement module for ambiguous instances and, conversely, the refinement process can be guided by retrieval errors. We aim to extend our framework beyond current TBPR benchmarks to broader cross-modal retrieval and person re-identification scenarios, including video-based person search, open-world surveillance with evolving identity pools, and other domains where imperfect, weak, or noisy textual descriptions remain a major bottleneck. Finally, our current work mainly studies mitigating annotation-induced mismatches through dataset refinement. In future research, we plan to further explore training methods utilizing MLLMs beyond dataset refinement, for example by incorporating an MLLM into the training loop to analyze hard negatives retrieved by a TBPR model and generate more discriminative supervision signals for improving cross-modal matching. Meanwhile, we will also explore MLLM-free alternatives, such as ambiguity discovery via text-only near-duplicate caption mining across different IDs, visually similar identity grouping with unsupervised ReID features, and attribute/part-based structured annotations with lightweight human verification, to reduce cross-ID caption overlap without relying on MLLMs. Limitations and future directions are discussed in Section 5. 

## Figures and Tables

**Table 1:** All possible scenarios between captions and images in the dataset.

Captions and Images in the Dataset	ID Relationship	
	Same ID	Different ID
Semantic Match	Evaluated as match; correct (1)	Evaluated as mismatch; error (2)
Semantic Mismatch	Evaluated as match; error (3)	Evaluated as mismatch; correct (4)

**Table 2:** Zero-shot discriminative capability on the modified portion of the CUHK-PEDES test set using frozen ViT-bigG-14 (CLIP) and SigLIP-large. CLIP_R@K, ambiguity rates, and relative changes are reported in %. MeanMargin is computed from cosine similarities and is reported as ×100 for readability.

ViT-bigG-14 (CLIP)				
**Metric**	**Before**	**After**	Δ **(After–Before)**	**Relative Change (%)**
CLIP_R@1	15.89	22.19	+6.30	+39.70
CLIP_R@5	32.12	39.61	+7.49	+23.30
CLIP_R@10	40.80	49.23	+8.43	+20.70
MeanMargin	−6.20	−5.09	+1.11	+17.90
Rate_margin <0	88.20	82.81	−5.39	−6.10
Rate_margin <0.01	91.93	87.62	−4.31	−4.70
SigLIP-Large				
Metric	Before	After	Δ (After–Before)	Relative Change (%)
CLIP_R@1	26.34	40.41	+14.07	+53.40
CLIP_R@5	44.61	60.72	+16.11	+36.10
CLIP_R@10	53.04	70.15	+17.11	+32.30
MeanMargin	−3.27	−1.61	+1.66	+50.70
Rate_margin <0	78.61	65.26	−13.35	−17.00
Rate_margin <0.01	85.49	75.04	−10.45	−12.20

**Table 3:** Human evaluation score statistics on 300 randomly sampled CUHK-PEDES query instances. Scores are on a 0–10 scale (higher is better). Each entry is formatted as mean/median/std; [Q25, Q75]. “Final” is the arithmetic mean of Naturalness and Useful&Faithful.

Metric	Original	Refined	Δ (Refined–Original)
Naturalness	7.26/7.0/1.78; [6.0, 9.0]	8.28/9.0/1.46; [8.0, 9.0]	+1.02/+1.0/2.49; [0.0, 3.0]
Useful&Faithful	5.64/6.0/2.18; [4.0, 8.0]	6.73/8.0/2.55; [4.75, 9.0]	+1.09/+2.0/3.81; [−2.0, 4.0]
Final (avg.)	6.45/6.5/1.54; [5.5, 7.5]	7.51/8.0/1.66; [6.0, 9.0]	+1.06/+1.5/2.49; [−0.5, 3.0]

**Table 4:** Human evaluation paired outcomes and statistical tests on CUHK-PEDES (N = 300). Pos/Neg/Zero denote the number of instances where the refined score is higher/lower/equal to the original score (with rates). Better labels are counts of {refined, original, tie, and both_bad}. “Win rate excl. ties” is refined/(refined+original) excluding ties with Wilson 95% CI. Mean Δ confidence intervals are reported for both bootstrap (5000 resamples) and normal approximation. The sign test is a two-sided exact sign test on non-zero score differences.

Metric	Pos/Neg/Zero (Rate)	Better Labels (R/O/T/B)	Win Rate Excl. Ties (Wilson95)	Mean Δ CI95 (Bootstrap)	Mean Δ CI95 (Normal)	Sign Test *p*
Naturalness	179/64/57 (59.7/21.3/19.0%)	177/64/59/0	73.44% [67.53, 78.62]	[0.737, 1.293]	[0.738, 1.302]	$8.88\times 10^{-14}$
Useful&Faithful	192/89/19 (64.0/29.7/6.3%)	190/88/15/7	68.35% [62.66, 73.53]	[0.643, 1.527]	[0.662, 1.525]	$7.60\times 10^{-10}$
Final (avg.)	194/87/19 (64.7/29.0/6.3%)	187/73/40/0	71.92% [66.17, 77.04]	[0.773, 1.327]	[0.775, 1.338]	$1.55\times 10^{-10}$

**Table 5:** Performance comparison on CUHK-PEDES before and after annotation refinement (ASM: After Separate Expansions Followed by Merging). All metrics are reported as percentages. Values in parentheses denote the absolute change (ASM–Original); red indicates improvements and green indicates decreases. TBPS-CLIP* denotes a simplified, lightweight variant of TBPS-CLIP used in our experiments.

Method	Modification	Rank@1	Rank@5	Rank@10	mAP
RaSa [22]	Original	76.12	89.90	93.65	70.26
	ASM	81.17 (+5.05)	90.58 (+0.68)	93.81 (+0.16)	72.94 (+2.68)
RDE [23]	Original	75.88	90.42	93.97	67.70
	ASM	80.34 (+4.46)	90.46 (+0.04)	93.63 (−0.34)	70.19 (+2.49)
TBPS-CLIP* [20]	Original	72.66	88.14	92.72	64.97
	ASM	79.34 (+6.68)	89.90 (+1.76)	93.16 (+0.44)	68.62 (+3.65)
IRRA [14]	Original	73.70	89.46	93.81	66.17
	ASM	79.37 (+5.67)	89.39 (−0.07)	92.58 (−1.23)	68.97 (+2.80)

**Table 6:** Performance comparison on RSTPReid before and after annotation refinement (ASM: After Separate Expansions Followed by Merging). All metrics are reported in percentage. Values in parentheses denote the absolute change (ASM–Original); red indicates improvements and green indicates decreases. TBPS-CLIP* denotes a simplified, lightweight variant of TBPS-CLIP used in our experiments.

Method	Modification	Rank@1	Rank@5	Rank@10	mAP
RaSa [22]	Original	66.45	85.25	90.85	52.09
	ASM	77.75 (+11.30)	91.05 (+5.80)	94.75 (+3.90)	57.38 (+5.29)
RDE [23]	Original	66.30	85.15	90.05	51.40
	ASM	75.15 (+8.85)	89.65 (+4.50)	93.55 (+3.50)	56.27 (+4.87)
TBPS-CLIP* [20]	Original	62.10	81.90	87.75	48.00
	ASM	69.10 (+7.00)	84.90 (+3.00)	90.85 (+3.10)	51.47 (+3.47)
IRRA [14]	Original	58.35	77.80	85.80	46.37
	ASM	67.45 (+9.10)	83.45 (+5.65)	89.35 (+3.55)	50.93 (+4.56)

**Table 7:** Performance comparison on ICFG-PEDES before and after annotation refinement (ASM: After Separate Expansions Followed by Merging). All metrics are reported in percentage. Values in parentheses denote the absolute change (ASM–Original); red indicates improvements and green indicates decreases. TBPS-CLIP* denotes a simplified, lightweight variant of TBPS-CLIP used in our experiments.

Method	Modification	Rank@1	Rank@5	Rank@10	mAP
RaSa [22]	Original	65.28	80.40	85.12	41.29
	ASM	66.15 (+0.87)	81.73 (+1.33)	83.86 (−1.26)	43.62 (+2.33)
RDE [23]	Original	66.83	82.16	86.81	39.83
	ASM	72.77 (+5.94)	85.03 (+2.87)	87.09 (+0.28)	45.47 (+5.64)
TBPS-CLIP* [20]	Original	64.52	80.03	85.39	39.54
	ASM	70.43 (+5.91)	81.22 (+1.19)	84.91 (−0.48)	42.36 (+2.82)
IRRA [14]	Original	62.37	79.23	85.26	38.26
	ASM	69.41 (+7.04)	78.93 (−0.30)	86.31 (+1.05)	40.19 (+1.93)

**Table 8:** Experiment on CUHK-PEDES with a recent advanced TBPR method (FCSA [15]) before and after annotation refinement (ASM). All metrics are reported in percentage. Values in parentheses denote the absolute change (ASM–Original); red indicates improvements and green indicates decreases.

Method	Modification	Rank@1	Rank@5	Rank@10	mAP
FCSA [15]	Original	75.07	89.72	93.73	69.56
	ASM	77.92 (+2.85)	88.52 (−1.20)	92.14 (−1.59)	71.30 (+1.74)

**Table 9:** Performance comparison of different caption refinement strategies for IRRA on CUHK-PEDES. All metrics are reported as percentages. Statistical significance validation of the differences in this table is reported in Table 10. The best results in each column are shown in bold.

Strategy	Rank@1	Rank@5	Rank@10	mAP
Original	73.70	89.46	93.81	66.17
Parallel Expansion	75.18	87.57	91.60	66.56
Serial Expansion	76.15	86.16	89.94	66.66
Separate Expansion Followed by Merging	**79.37**	**89.39**	**92.58**	**68.97**

**Table 10:** Statistical significance validation for Table 9 on CUHK-PEDES (IRRA). Delta is defined as Separate Expansion Followed by Merging minus Baseline (percentage points). Rank@K uses the exact two-sided McNemar test; mAP uses the paired *t*-test with paired bootstrap 95% confidence intervals (20,000 resamples) and a paired sign–flip permutation test (20,000 permutations).

Baseline	Rank@1		Rank@5		Rank@10		mAP			
	Δ	p	Δ	p	Δ	p	Δ	CI95%	t	$p_{t}$
Original	+5.669	$5.85\times 10^{-36}$	−0.065	0.9027	−1.235	$2.00\times 10^{-4}$	+2.804	[2.324, 3.292]	11.413	$7.17\times 10^{-30}$
Parallel Expansion	+4.191	$8.75\times 10^{-21}$	+1.819	$6.60\times 10^{-6}$	+0.975	0.0071	+2.404	[1.919, 2.895]	9.649	$7.09\times 10^{-22}$
Serial Expansion	+3.216	$6.66\times 10^{-13}$	+3.233	$5.24\times 10^{-15}$	+2.632	$2.43\times 10^{-12}$	+2.304	[1.792, 2.815]	8.809	$1.62\times 10^{-18}$

**Table 11:** Quantitative comparison of different MLLMs under the rewriting setting on CUHK-PEDES (IRRA). All metrics are reported as percentages. The best results in each column are shown in bold.

MLLM	Rank@1	Rank@5	Rank@10	mAP
IDEFICS	57.89	75.50	82.31	53.83
LLaVA v1.6	60.80	**77.07**	**83.41**	55.60
Qwen-VL	**63.32**	76.66	82.53	**56.78**

**Table 12:** Sensitivity analysis of AmbiguousRate(τ) on the modified portion of the CUHK-PEDES test set under different thresholds τ using frozen ViT-bigG-14 (CLIP) and SigLIP-large. All metrics are reported as percentages.

τ	ViT-bigG-14 (CLIP)		SigLIP-Large	
	Before	After	Before	After
−0.05	56.77	47.98	31.90	18.21
−0.02	78.19	71.01	59.87	44.91
−0.01	83.91	77.25	69.62	55.06
0	88.20	82.81	78.61	65.26
0.01	91.93	87.62	85.49	75.04
0.02	95.02	91.51	90.38	82.53
0.05	98.87	97.84	97.40	95.11

## Data Availability

Data available in a publicly accessible repository. The improved annotations of these benchmarks will be released publicly at https://github.com/hzh0720/Revisiting-Text-Based-Person-Retrieval, accessed on 2 March 2026.
